# Movement Patterns of Roaming Companion Cats in Denmark—A Study Based on GPS Tracking

**DOI:** 10.3390/ani12141748

**Published:** 2022-07-07

**Authors:** Helene Ane Jensen, Henrik Meilby, Søren Saxmose Nielsen, Peter Sandøe

**Affiliations:** 1 Department of Veterinary and Animal Sciences, University of Copenhagen, DK-1870 Frederiksberg C, Denmark; scf887@alumni.ku.dk (H.A.J.); saxmose@sund.ku.dk (S.S.N.); 2 Department of Food and Resource Economics, University of Copenhagen, DK-1958 Frederiksberg C, Denmark; heme@ifro.ku.dk

**Keywords:** domestic cat, *Felis catus*, GPS tracking, habitat, home range, rainfall, roaming

## Abstract

**Simple Summary:**

In comparison with other companion animals, domestic cats are more likely to roam freely, and this can give rise to conflicts and controversies. To assess the potential magnitude of the problems posed by free-roaming companion cats it is important to know how large an area outside their owners’ property they typically cover. Using GPS tracking, we studied nearly 100 cats in Denmark, a temperate country where around 14% of families own one or more cats, of which nearly three quarters are allowed to roam. We found that although the majority of the cats spent most of their time at their owner’s property and were less active when it rained, they still roamed a lot. The middle value (median) of the area used by the cats was the size of around seven soccer fields (5 ha), but there was huge individual variation, ranging from a little over one to over 150 soccer fields (1–113 ha). Thus, in a suburban neighbourhood, a free-roaming companion cat will typically pass through a lot of other people’s gardens. The area in which the cats roamed tended to be larger when they were younger, had access to nature areas, or were intact males.

**Abstract:**

We studied the roaming patterns of companion cats in Denmark. The movements of 97 cats with outdoor access were traced for about seven days using GPS tracking. Data on the cats were gathered from their owners. The median time cats spent away from their homes was 5 h per day (IQR: 2.5 to 8.8 h), median daily distance moved was 2.4 km (IQR: 1.3 to 3.7 km), and median for 95% BBKDE home range was 5 ha (IQR: 2.9 to 8.5 ha). Cats above seven years of age spent less time away from home, were less active and had a smaller home range than younger cats. Cats with access to nature areas spent more time away from home, were more active and had larger home ranges. Intact male cats spent more time away from home than neutered cats and had larger home ranges as well. Finally, rainfall had an impact on the distance moved by cats: on days without rainfall the cats moved 3.6 km on average (95% CI: 2.8; 4.5 km); and on days with heavy rainfall the cats moved 2.4 km on average (95% CI: 1.6; 3.5 km).

## 1. Introduction

Domestic cats (*Felis catus*) are carnivores and predators and are at the same time among the most popular pet animals. Approximately 370 million domestic cats are estimated to be kept as companion and utility animals globally [1]. Compared with other companion animals such as dogs, cats have a special status in that they more often roam freely without their owners’ supervision, potentially giving rise to conflicts and controversies. In the United States, United Kingdom, Australia, New Zealand, and other countries, there is an ongoing debate about whether cats should be allowed to freely roam outdoors [2]. Concerns about the ecological impact of cats on wildlife populations and native species, feline transmission of zoonotic diseases, and the inconvenience that roaming cats might cause to other people have led to various suggested initiatives, including night curfews, collars with bells, cat-free buffer zones around nature reserves or sensitive conservation areas, and routine confinement [3,4,5,6,7,8,9,10]. Another factor supporting indoor confinement is the concern that free-ranging cats may be injured by other cats, humans, cars or predators (e.g., feral dogs, coyotes, wolves) [11]. On the other hand, some disapprove of the indoor confinement of cats because it has been associated with an increase in behavioural disorders [12] and with health problems such as obesity, diabetes mellitus type 2, and urinary tract disease [13,14,15]. As well as serving as companions to families, cats can have a utility role. Domestic cats have traditionally been viewed as useful for rodent control on farms and other locations, but the actual effect is disputed [16]. These contrasting opinions on advantages and drawbacks can turn into significant anti-social disputes as cats roam freely on the properties of neighbours, preying on wildlife, defecating in other people’s gardens, and fighting with other people’s cats [17].

In Denmark, the public debate is not nearly as pronounced as in the countries mentioned above. In 2021, it was estimated that there were around 730,000 companion cats in the country residing in 14% of Danish families [18]. In a survey of Danish cat owners conducted in 2015, 72% reported that their cats had outdoor access [19]. The prevalence of cat ownership in Denmark is relatively low in comparison with other countries. For example, in the USA and the United Kingdom 25–27% of households have been reported to own at least one cat [20,21]. The conservation debate over cats currently running in other countries also seems less prominent in Denmark, but there are still divided opinions about free-ranging cats in the country. A 2015 questionnaire survey revealed that one in four Danes considered free-ranging cats to be problematic, with feline defecation in gardens being the main concern [19]. Even in Denmark, parties with concerns about wildlife argue that domestic cats present a threat to terrestrial birds, reptiles, and bats. Despite these concerns, the degree to which free-ranging cats threaten native wildlife through predation is unknown.

The impact of cats on their surroundings has been investigated by counting the prey items they bring in [2,22,23,24,25,26], by direct visual inspection or camera surveillance of the cats’ activities [27,28,29], and by feline scat counts [23,30,31]. The stomach contents of cats have also been examined [23,32] and cats have been tracked via radiotelemetry or GPS [4,6].

To measure how far companion cats actually move in conditions like those in an advanced, prosperous, temperate country such as Denmark, accurate tracking is required. Time-sensitive spatial data can be used to assess how much time cats spend outside, the distances they roam, and their home ranges, i.e., the areas that they most often reside in and typically traverse while roaming [26]. The most common method used to understand the movement patterns of domestic cats is the estimation of home ranges. Home ranges have been estimated in a number of countries beyond Denmark, including Sweden [27], Norway [33,34], France [24], the United Kingdom [2,8,10,35], Switzerland [36], Corvo Island [31], the United States [2,30,37,38,39], Africa [40], Australia [2,4,7,17,41], and New Zealand [2,5,6,22,26,29,42,43].

The vast literature indicates that cats’ home ranges vary as a function of: (1) geographical region; (2) habitat characteristics; (3) sex and neuter status; (4) age; (5) cat population density; (6) resource (food) abundance; and (7) the presence of predators [6,9,22,44,45,46]. For example, home ranges may be larger for male cats and for cats less than eight years of age (reviewed in [44]). Additionally, in some rural areas with low cat population density, cat home ranges were found to be larger than those of cats living in some suburban areas with higher cat population densities [2,4,10,22]. It is noticeable, however, that the results of these studies vary. The fact that the home range estimates differ from study to study is interesting in itself. It could suggest that the spatial needs of domestic cats are not yet fully understood. The literature indicates that there are large individual differences between cats’ spatial needs. What we do know is that the range of findings, combined with small sample sizes and different methods of collecting and analysing spatial data [47], makes it difficult at present to draw firm conclusions about the factors affecting home range size in roaming cats.

Cats roam to find mates (when intact), to explore, and to forage [46]. As opportunistic feeders, domestic cats have varying activity patterns depending on the availability of food. Although some cats still hunt after eating food provided by their owners, hungry cats with restricted access to owner-provided food might spend more time hunting than cats with ad libitum access to food [46,48].

Cat movements can be restricted by their owners [33], by barriers such as infrastructure and busy roads [4], and by rainfall [7,35,49]. Cats with restricted outdoor access may roam less than those that are strictly outdoor cats or those left to roam as they please via permanently open doors, windows, or cat flaps.

The effect of cat breed is at present under-investigated [17,26], but it may be a factor given that some breeds, such as Persian cats, tend to be less active [46]. As to the effect of neutering, a meta-analysis from 2016 found no significant effect on home range [44], but there are newer studies, which conclude that intact male cats range further than neutered cats [2,47].

In Denmark the majority of companion cats (86%) are neutered [12]. As a result, they may roam less because they no longer have the urge to search for a mate, and only roam to explore and hunt [46]. Furthermore, Danes live fairly close to each other, and there are few predators that present a threat to cats in the country. These are all factors that could influence the roaming of Danish domestic cats, creating contrasts with the findings reported in some other countries. Additionally, studies have found evidence that cats prefer to visit nature areas [6,22,33,37]. They may, therefore, trek an additional distance to reach their preferred roaming areas.

The objectives of the study were to measure and compare: (1) the time companion cats spend away from their homes; (2) the daily distance moved at different levels of rainfall; and (3) home range estimates as a function of (a) sex and neuter status, (b) age, (c) breed, (d) degree of outdoor access, (e) access to food, (f) land use (rural, suburban, summer cottage, urban, or industrial), (g) nearby presence of busy roads, and (h) access to nature areas. Movement and associated data were gathered using GPS collars and questionnaire surveys.

## 2. Materials and Methods

### 2.1. Study Area

This study followed a cohort of companion cats with outdoor access for seven days. Both the method of tracking the cats and the survey were approved by two review boards, one for animal ethics and one for human research ethics, serving the relevant faculties at the University of Copenhagen (see section ‘Institutional Review Board Statement’). The data were collected between 30 July and 30 November 2021. In this period, the mean temperature was 11.9 °C and ranged between −5.2 °C and 26.9 °C. The country’s average total precipitation per month was 75 mm and ranged between 54.5 mm and 99.4 mm per month.

Using the relevant GPS data, together with SDFEs Map Viewer (The Agency for Data Supply and Efficiency, Copenhagen, Denmark) and the land cover classes provided by the CORINE Land Cover project (Coordination of Information on the Environment; European Union, Copernicus Land Monitoring Service 2018, European Environment Agency), the cats’ habitats were divided into three categories: urban and industrial areas (continuous urban fabric); suburban and summer cottage areas (discontinuous urban fabric and sport and leisure facilities); and rural areas (land principally occupied by agriculture, with significant areas of natural vegetation, and non-irrigated arable land).

Data on daily rainfall in the municipalities where the cats were monitored were collected from the weather archive of DMI (Danish Meteorological Institute, Copenhagen, Denmark). For each cat, the tracking period and name of municipality were selected to retrieve information on the amount of rainfall in mm per day.

### 2.2. GPS Tracking

#### 2.2.1. Recruitment of Cat Owners

Cat owners were recruited through social media and separately with the assistance of veterinary students who participated in the study with their cats or solicited acquaintances. Only companion cats with outdoor access and above one year old were included in the study.

#### 2.2.2. Data Collection

We collected movement data by fitting cats with GPS trackers (Tractive GmbH, Pasching, Austria). The trackers had a battery life of two to five days and were waterproof and shock resistant. Each device weighed 28 g and measured 28 mm × 74 mm × 15 mm. As a default, the tracker was mounted on a collar with a self-releasing safety buckle to reduce risk of injury or entanglement. The tracker and collar together weighed 43 g.

The GPS tracking system recorded locations every 2–60 min, depending on the activity levels of the cats: the trackers had accelerometers and collected GPS locations every 2–10 min when the cats were moving, and once every 60 min if the cats were inactive. Recordings were more frequent (1 to 2 s intervals) if owners activated the live-tracking mode. The intervals of the GPS fixes were also affected by interference caused by, for example, the cats being indoors, or in densely built-up areas or in dense forest areas. Such interference resulted in fewer fixes. The mean location error of ten tracking devices placed in different environments for 48 h was 13.1 m with a standard deviation of 8.7 m (see summary of location errors in Supplementary Materials S2). Coordinates were calculated by cell tower triangulation [50].

Owners received instructions on how to use, charge and fit the GPS collars to their cats. Some reported problems with collars that fell off too easily (e.g., if the cat scratched itself). For this, we devised two possible solutions: (1) A piece of tape could be placed in the closing mechanism of the safety collar to improve its binding ability; and (2) a collar with a buckle clasp was offered as an alternative to cats that repeatedly removed the collar. The second collar did not have a self-releasing safety mechanism and if owners chose this solution, they were advised to be aware of the movements of their cat to reduce the risk of entanglement. The collar had an attached bell that was removed. With each animal, both the owner and the first author, H.A.J., were able to follow the movements of the cat on a mobile app.

Where cats were unaccustomed to a collar, the owners were asked to make sure that their cats had an adaptation period of one to five days after which they (the owners) would evaluate whether or not the cat had accepted the collar. The aim was to ensure that all cats were accustomed to wearing a collar before they were let out to collect GPS data. Six cats that did not habituate to the collar within the adaptation period were excluded from the study.

#### 2.2.3. Data Management

GPS data were downloaded from Tractive’s website as GPX files, which were opened in Garmin Basecamp (Garmin, Lenexa, KS, USA) and converted to CSV files. All first coordinates were made equal to the home address of the cat to discriminate between ‘home’ and ‘away’. Observations recorded with a speed of movement between locations exceeding 5–10 m/s were removed from the dataset, depending on the general speed of the cat; the upper speed limit was set to 10 m/s for cats that had several data points between 5–10 m/s but few very high outliers above 10 m/s; the upper limit was set to 5 m/s for cats that had few very high outliers above 5 m/s. These limits were set arbitrarily but in line with the observed data. Thus, these observations were removed to exclude GPS errors from the datasets and to avoid overestimation of home ranges in later data analysis. This data cleaning was performed in Excel (Microsoft, Redmond, WA, USA).

### 2.3. Questionnaire Survey

#### 2.3.1. Data Collection

Cat owners were asked to complete a questionnaire with up to nine questions (see questionnaire in Supplementary Materials S1). The precise number of questions asked depended on the previous answers given. The questionnaire was distributed to the owners on paper or online via SurveyXact (Rambøll Management Consulting, Aarhus, Denmark). Its purpose was to obtain relevant information about the cats, which could not be obtained from other data sources.

#### 2.3.2. Study Variables

The following information about the cats was obtained from the responses to the questionnaire: sex and neuter status; age (divided in three almost equally sized groups, with 1–3 years representing young cats, 4–7 years representing adult cats, and >7 years representing mature adult and senior cats); breed (domestic shorthair, purebred, mixed/unknown breed); degree of outdoor access (presence of cat flap, and day or night restrictions); access to food (ad libitum or restricted); presence of larger busy roads within 300 m of the cat’s home; and access to nature areas within 1 km of the cat’s home.

### 2.4. Time Spent Away from Home Base

A cat’s ‘home base’ was defined as the area within a radius of 50 m from their home address. The time spent within 50 m of the first recording was thus considered as ‘being at home base’, while the remaining time was considered ‘away’. This was to account for inaccurate recordings of locations when cats were indoors and to not overestimate time spent ‘away’. The radius of 50 m was chosen with reference to the location errors of tracking devices (the average location error was 13.1 m with a standard deviation of 8.7 m) and the mean housing density of a typical suburban property (700–1000 m^2^). Defining the home radius as 50 m therefore ensured that cats described as being ‘away’ were in most cases outside their owners’ property.

### 2.5. Distance Moved

GPS data were used to calculate the daily distance traversed by each cat. The distance moved was calculated by summing the distances measured between recorded locations. To reduce the risk of underestimation here, data from first and last tracking days were removed, because the cats were typically not tracked for the entire first and last days of tracking.

### 2.6. Home Range Estimates

Home ranges were calculated by 95% Brownian bridge kernel density estimation (95% BBKDE) [51,52,53]. Duplicate locations were removed during data quality control. The estimates were calculated in R (R Core Team, Vienna, Austria) with the package adehabitatHR and the kernelbb function [54].

### 2.7. Statistical Analysis

All data were analysed using the statistical software R (version 4.1.2, R Core Team, Vienna, Austria). We tested the association between the explanatory variables using Pearson’s Chi-squared test and excluded variables that were strongly associated with other variables [55].

We attempted two approaches to modelling the percentage of time cats spent away from their home base: a linear model with cubic root transformation and a beta regression model. Using the Akaike information criterion (AIC) to rank the modelling approaches, we found that the beta regression model produced lower AIC values and therefore we proceeded to use this approach to modelling. We used the betareg-package in R [56], and both the logit, log and probit links were tested. The logit link function appeared better based on the pseudo-R-squared. Therefore, we used the logit link and assumed identity link for phi. The full model with all variables was reduced using manual backward elimination. We tested the significance of the variables by analysis of variance (Anova type II) [57] with a 5% level of significance, while also assessing the effect of confounding.

The effect of rainfall on the distance moved by the cats was analysed in a linear mixed model [58] using the lme-function in the nlme-package [59]. The data were transformed using Box–Cox transformation with the boxcox-function in the MASS-package [60], because of their skewed distribution. Cat ID was included as a random effect to take into account the repeated measurement of cats over several days. Autocorrelation was addressed by assuming a first-order autoregressive process (AR1), which improved the model based on AIC. Variables were selected for the model by manual backward elimination. We tested the significance of the variables by Anova type II with a 5% level of significance, while also assessing the effect of confounding. Predictions for the effect of rainfall were estimated using the function ggpredict from the ggeffect-package [61].

The associations between home range and the variables of interest were tested by analysis of variance using the functions lm and Anova type II with a 10% level of significance. To meet the assumption of normality, the home ranges were log-transformed. Variable selection was performed on the full model using manual backward elimination. Three assumptions were made: independence of observations, homogeneous variance, and normal distribution of the residuals. Post-hoc testing was performed for pairwise comparison of significant values using the emmeans-package [62]. Each significant variable was tested for confounding, i.e., its sensitivity to the inclusion of other variables in the model. If a parameter estimate changed by >20% when including another variable in the model, the variables were considered confounded.

## 3. Results

For all analyses, the explanatory variables Restrictions to outdoor access and Type of outdoor access were removed due to their strong association with other explanatory variables.

### 3.1. Population of Cats

Ninety-seven cats with ages ranging between 1–16 years were included in the study (Appendix A) (GPS data from 59 of the cats are included in the data analysed in [63], also published in *Animals* 2022). Of these, 49 were male (four intact, 45 neutered) and 48 were female (three intact, 45 neutered). The veterinary students of year 2021 provided data from 27 of these cats. Eight of the included cats already used a Tractive GPS. Although the participating cats were in general tracked for seven days (median = 7.0, mean = 7.1, and standard deviation = 1.1), three cats were tracked for six days (Appendix A). Four cats had an extended tracking period due to lost trackers that interrupted the data collection, but disregarding the interruptions these cats were also tracked for approximately seven days. Three cats were excluded because they were only tracked for one to three days before their owners reported that the trackers had become lost and that they were unable to find them. No owners reported cats being stuck or injured as a result of wearing the collar.

**Figure 1:**
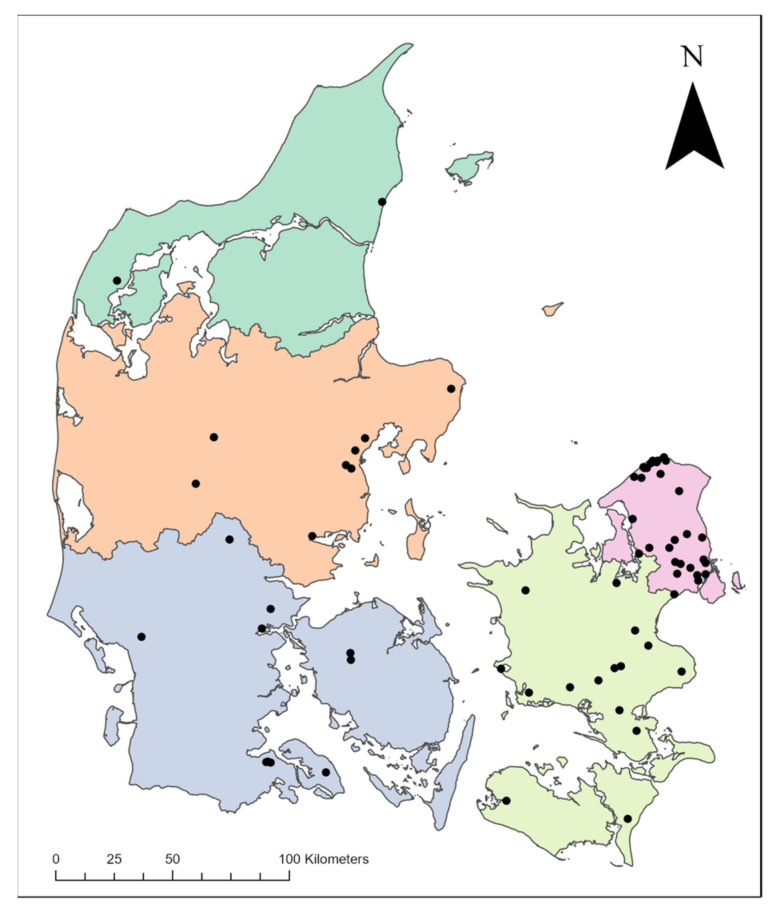
Geographical distribution of the 97 companion cats tracked in Denmark. Based on map data provided by The Danish Agency for Data Supply and Efficiency, Copenhagen, Denmark, and Danish Municipalities; ‘DAGI’ (downloaded 08/2020).

Of the cats, 12 were from urban or industrial areas, 31 lived in rural areas, and 54 were from suburban or summer cottage areas (where the mean housing density for the suburban areas was 1150/km^2^ (868 m^2^ per property) and the mean housing density for the summer cottage areas was 624/km^2^ (1602 m^2^ per property). The geographical distribution of the 97 cats is shown in Figure 1. Cats from all five regions in Denmark were represented: 36 from the Capital Region of Denmark; 25 from the region Zealand; 21 from the region of Southern Denmark; 12 from the Central Denmark region; and three from the North Denmark region. The cats were distributed across 36 of the 98 municipalities in Denmark (Appendix A).

### 3.2. Time Spent Away from Home Base

The median time cats spent away from their home base was 5.16 h per day, with a minimum of 0.37, an interquartile range (IQR) of 2.49 to 8.77, a maximum of 21.03 and a mean of 6.15 h. In the full model describing percentages of time spent away from home base, the variables Age group and Access to Nature areas were significant at the 5% level. The full model was reduced by manual backward elimination and the reduced model shown in Table 1 included Sex, Age group and Access to Nature areas. The differences in time spent away from home base between sexes were significant at the 10% level (*p* = 0.06), and intact male cats tended to spend more time away from their home base than neutered males and females. The differences between age groups were significant (*p* = 0.004), and cats above 7 years of age spent less time away from their home base than those in both of the groups of younger cats in the post-hoc test. The variable Access to Nature areas was also significant (*p* = 0.01), and cats with access to nature areas spent more time away from home base than cats without access to nature areas. The estimate for Access to Nature areas changed by more than 20% when models with and without Age group were compared, strongly suggesting confounding variables. The estimate for Sex also changed by more than 20% when adding Access to Nature areas to the model.

### 3.3. Distance Moved

The distances moved by 95 of the cats were analysed (Cats 9 and 43 were excluded because of interruptions in daily data collection). The median daily distance moved was 2.37 km, with a minimum of 0.08, an IQR of 1.34 to 3.68, a maximum of 9.36 and a mean of 2.75 km. The Box–Cox transformation suggested a lambda of 0.34 and the data were transformed accordingly.

By manual backward elimination, the variables Rainfall (*p* = 0.01), Access to Nature areas (*p* < 0.001), Age group (*p* < 0.001) and Breed (0.02) were selected for the model presented in Table 2, and the variables were significant at the 5% level. Cats above 7 years of age were less active than those in the two younger age groups in the post-hoc test, and cats without access to nature areas were less active than cats with such access. Finally, domestic shorthaired cats moved less than mixed/unknown breeds and purebred cats (i.e., Bengal, Birman, Maine Coon, Norwegian Forest cat, Ragdoll, and Russian Blue). When the variables were tested for confounding factors, Access to Nature areas, Age group and Breed were found to be sensitive to the inclusion of other variables (estimates changed by >20%).

In Table 3, the estimates for cats’ distance moved on days with different levels of rainfall are adjusted for the significant variables and autocorrelation. The predicted distance moved on days with heavy rain was significantly less (mean = 2.4 km) than the distance on days without rainfall (mean = 3.6 km, *p* = 0.01).

### 3.4. Home Range Estimates

#### 3.4.1. Descriptive Statistics

The estimated 95% BBKDE median home range of the 97 cats was 5 ha, with the distribution shown in Table 4. The home range estimate of each cat is shown in Appendix A.

The median home range of neutered male cats was 18% larger than that of neutered females. Intact males had the largest home range estimates, but the sample size of intact male cats was small (*n* = 4) in comparison with the sample of neutered cats (45 males and 45 females). Home range estimates shrank with increasing age, while only minimal differences emerged between breeds and types of outdoor access. Cats that were out only at night and those with restricted access to food appeared to have larger home ranges, as did those living in rural areas and those without busy roads near their home. Finally, the median home range of cats with access to nature areas appeared larger than that of cats without access to such areas.

#### 3.4.2. Analytical Statistics

The home range estimates were logarithmically transformed and three outliers with home ranges above 90 ha were excluded (Cats 71, 77 and 78) to ensure normality. The remaining cats had home ranges below 40 ha.

In the full model, including all variables from Table 4, Age group was significant at the 5% level and the variables Access to Food and Access to Nature areas were significant at the 10% level. The remaining variables from Table 4 were non-significant. Using manual backward elimination for variable selection, the variables Sex, Age group and Access to Nature areas were included. In the final model, differences in home range between age groups were significant (*p* < 0.002) and the variables Sex and Access to Nature areas were significant at the 10% level (*p* = 0.086 and *p* = 0.077, respectively). The estimates for the variables Access to Nature areas and Sex changed by more than 20% when they were separately compared in models with and without Age group, and therefore confounding was strongly suggested. Home range size declined with increasing age; cats with access to nature areas had larger home ranges; and although the sample size of intact males was small, intact male cats tended to have larger home ranges as well (see Table 5). The assumptions of independence, and of normally and identically distributed residuals, were assessed to be fulfilled. Parameter estimates of the final model with the variables Sex, Age group and Access to Nature areas are shown in Table 5.

## 4. Discussion

In summary, the median time cats spent away from their home base was 5 h per day (IQR: 2.5 to 8.8 h), the median daily distance moved was 2.4 km (IQR: 1.3 to 3.7 km) and the median for 95% BBKDE home range was 5 ha (IQR: 2.9 to 8.5 ha). Cats above seven years of age spent less time away from their home base, were less active and had a smaller home range than cats less than seven years old. Similarly, cats with access to nature areas were more active and had larger home ranges than those without such access. Intact male cats (*n* = 4) also had larger home ranges. Domestic shorthaired cats were less active than those of mixed/unknown breeds and purebreds. Finally, rainfall had an impact on the distance moved by the cats: on days without rainfall the cats moved 3.6 km on average (95% CI: 2.8; 4.5 km); and on days with heavy rainfall they moved 2.4 km on average (95% CI: 1.6; 3.5 km). The remaining variables assessed were non-significant.

As regards the concerns about free-ranging domestic cats mentioned in the Introduction above, our study confirms that free-ranging cats may pose a risk to wildlife in nature areas located close to areas where there are humans who keep free-ranging companion cats. However, since domestic cats have been abundant in Denmark for more than 1000 years [64], it could be argued that most local wildlife should have adapted to predation pressure from these animals. Our study also shows that free-ranging cats are likely to traverse a lot of gardens in suburban areas. Such cats are potentially hazardous transmitters of zoonotic diseases, such as toxoplasmosis via faeces in sandboxes, and are in various ways likely an inconvenience to people who do not like cats.

### 4.1. Findings in the Light of Other Studies

Of the three outcome variables analysed, home range is the one most often used to describe the movement patterns of cats in the existing literature. There is considerable variation in estimates of the home ranges of companion cats between studies, with a range from below 1 ha to around 300 ha [4,39]. In New Zealand, a study reported home ranges from 0.1 ha to 213.9 ha (mean = 3.28 ha, median = 1.3 ha, *n* = 209) [26]. In a rural area in New Zealand, the median home range of 13 cats was 18 ha, while the median home range of 25 cats in urban areas was 1 ha [6]. In Northwest Georgia, USA, farm cats tracked during different seasons had mean home ranges from 4.26 ha (spring, *n* = 7) to 10.23 ha (winter, *n* = 5) [30]. A larger home range of 155 ± 40 ha (mean ± SD) was found for 11 farm cats in Illinois, USA [39]. In a study from Canberra, Australia, the home range of ten suburban cats was 2.73–7.89 ha (mean diurnal home range–mean nocturnal home range) [4]. In urban Perth, Australia, the mean home range (±SE) of 34 cats was 2.17 ± 0.82 ha [7], and in a national park in Bherwerre Peninsula, Australia, the mean home range (±SE) of 15 cats was 2.92 ± 1.13 ha (median = 1.5 ha) [41]. Urban home range was estimated to be larger in a Master’s dissertation from Cape Town, South Africa, where 14 cats from deep-urban and urban-edge areas had a mean home range of 31.65 ha [40]. These home ranges were estimated using 100% MCP (Minimum Convex Polygon) and were based on GPS monitoring for five to ten days. The use of the 100% MCP method may overestimate home range, as all observations and possible errors are included in it, while tracking of cats for ten days may indicate that the entire home range was revealed. Smaller home ranges were reported in a study from France where the mean home ranges (±SE) were 3.5 ± 0.3 ha for nine rural cats, 2.1 ± 0.2 ha for nine suburban cats, and 1.4 ± 0.1 ha for 12 urban cats [24]. In Reading, United Kingdom, 38 cats from different degrees of urban areas had a median home range of 1.28 ha [10]. A Master’s dissertation from Ås, Norway, reported a mean home range (±SE) of 3.57 ± 1.43 ha for 11 suburban cats [33]. A different Norwegian Master’s dissertation reported median home ranges of 1.5 ha for 85 urban cats and 4.3 ha for 19 rural cats [34].

Although the home range sizes in these studies vary, they do indicate that rural cats have larger home ranges than suburban cats, and that suburban cats have larger home ranges than urban cats [4,6,9,10,25,37,41], a pattern broadly correlating with the findings of smaller home ranges in areas with high cat densities [2,22,44,45]. We observed a similar trend, while 31 cats from rural areas (land principally occupied by agriculture, with significant areas of natural vegetation, and non-irrigated arable land) had a median home range of 5.60 ha (mean = 16.8 ha), 54 cats from suburban/cottage areas (discontinuous urban fabric and sport and leisure facilities) had a median home range of 5.02 (mean = 6.63 ha), and 12 cats from urban/industrial areas (continuous urban fabric) had a median home range of 3.43 ha (mean = 5.20 ha). However, the differences were not statistically significant due to major variation within groups.

When comparing home ranges in Denmark to those reported in other studies it is important to note that different methods for calculating home ranges are used in the extant studies, which are based on differing sample sizes and use various tracking periods. Sample sizes range from eight cats to 875 cats [2], and tracking periods down to one and a half days have been used [24]. With the different tracking methods (radiotelemetry or GPS) and frequencies of recorded locations, it is possible that in some studies entire home ranges may not be revealed [29,39,43]. Another issue is that many studies report the mean instead of the median. As Table 4 shows, there is often a considerable difference between mean and median due to outliers. The median should be reported because it is more resistant to these outliers.

In relation to possible explanatory variables for home range, a meta-analysis from 2016 combined smaller studies and found male cats to have significantly larger home ranges than females, but the effect of neutering on home range was non-significant [44]. Other studies, however, have found significantly larger home ranges for intact males [2,30,47]. In the present study, the difference between the home ranges of neutered male (median = 5.23 ha) and female (median = 4.44 ha) cats was non-significant. Sampling of intact companion cats in Denmark has proven difficult because 86% of Danish cats are neutered [12], and this is reflected in the current study (four intact males and three intact females in the sample). Nonetheless, we found intact male cats to have a much larger home range (median = 15.72 ha) than both neutered cats and intact females (median = 2.04 ha, see home range estimates for each cat in Appendix A). Therefore, this study supports previous studies in suggesting that neutering may reduce the home ranges of male cats due to a possible switch of interest from females to food [4,45].

Consistently with the results of this study, others have found age to have an impact on home range, with older cats having smaller home ranges than younger ones [2,25,31,34,35,44]. Few studies have shown otherwise [10,26]. Likewise, other studies have found that home ranges were larger when nature areas such as agricultural land, forest and areas of natural vegetation were accessible near to the buildings in which the cats were homed [6,7,22,24,33,37]. In line with a study from Poland [49], but unlike a study from the United Kingdom, which in fact had too few observations (*n* = 10) to draw any conclusions on the effect of rainfall [35], our study found cats to be significantly less active on days with rainfall. The effect of breed on home range was also non-significant in studies from South Africa (*n* = 428) and New Zealand (*n* = 209) [17,26], but we found breed to be associated with daily distance moved. Our finding that the purebred cats tended to be more active than the domestic shorthaired cats may reflect the inclusion of breeds such as Bengals and Maine Coon cats, as these have been described as high activity level breeds [65]. Barriers such as busy roads limited the size of home range in the study from Canberra [4], but the impact of busy roads was not significant in the current study. This may be due to differing assumptions about what counts as a ‘busy road’ made by cat owners. The cats in the New Zealand study spent more time away from home (median = 12 h) [26] than those in this study (median = 5 h). Possible explanations of this include different housing densities and thus cat densities, and that most cats in this study had ad libitum access to food in their homes and therefore may not have been dependent on searching for food and shelter as they can rely on their owners for such resources. Furthermore, 31 cats in this study had restricted outdoor access, and this may have had an effect on the percentage of time the cats spent away from their owner’s property.

### 4.2. Strengths and Limitations of the Study

#### 4.2.1. Strengths

The strengths of this study (in no particular order) are its relatively large sample size, the period the cats were given to become accustomed to the collar before data were collected, and the combination of GPS data, a questionnaire survey, and other data sources (DMI, SDFE). Furthermore, the study sample contained cats from both urban, suburban, and rural areas, unlike other studies where rural cats are not represented [10,40,47]. The home ranges were calculated using 95% Brownian bridge kernel estimation, which accounts for spatial dependence between locations. Other studies have estimated home ranges using other methods, such as minimum convex polygon, which may include unused areas or exclude areas known to be used by the cats [52].

#### 4.2.2. Limitations

The present study also had certain weaknesses, and the potential impact of these on the results will now be discussed. Our cat owners were recruited by convenience sampling through social media, and 27 cats were identified mostly via acquaintances of the veterinary students. The sample was therefore not necessarily representative of the population of companion cats in Denmark as a whole. Owners may have felt encouraged to participate because they had active cats, or cats that were easy to handle and likely to accept a collar, leading to a degree of overrepresentation of such cats. Another limitation is the small sample of cats from urban/industrial areas.

The GPS tracking period of six to seven days may not accurately reveal the entire home ranges of the cats, but this error is considered to give rise to non-differential misclassification bias only. One study recommended a tracking period of six days [22], while another found that variance in home ranges decreased after five days of tracking and was estimated most precisely after ten days [2]. The cats in our study were acclimatised to the collar for one to five days before data collection. This reduced the risk that they would alter their peripatetic behaviour as a result of wearing the collar, perhaps because of the weight of the device. However, it remains the case that a longer period of tracking would have given a better representation of home ranges. The GPS devices contain limitations as well: errors in recorded locations were recurrent (see location errors of tracking devices in Supplementary Materials S2), especially in densely built areas. This may have led to overestimation of the home ranges.

Turning to the questionnaire survey, we note that the responses to this may have contained inaccuracies. For example, mistakes may have appeared in owners’ subjective evaluations of whether busy roads were present within 300 m of their homes and whether there was access to nature areas within a 1 km distance of their homes.

In relation to data management, the number of observations was reduced to calculate the 95% BBKDE home ranges. This emphasises the need for more observations in the form of longer tracking periods. Data grouping can import limitations as well, as the same results may well not be obtained when data are grouped differently. In our study, cats from urban and industrial areas were grouped, as were those from suburban and summer cottage areas, on the basis that the areas grouped in this way were similar in their density of housing and degree of intensively used land. The age groups we used were assessed as reasonable classifications of the biological life stages of cats, i.e., 1–3 years representing young cats, 4–7 years representing adult cats, and >7 years representing mature adults and senior cats.

The R-squared and pseudo-R-squared values for the models in Table 1, Table 2 and Table 5 were rather low (0.25, 0.59, and 0.23, respectively). This may indicate that the dependent variables were influenced by other factors that are not accounted for in the models, such as the weight of the cats, their activity level, and individual preferences. A seasonal aspect may also lead to uncertainty about home range estimates. The cats in the present study were tracked from July to November. In France, suburban cats have been found to have a larger home range during April to June [24]. On the other hand, temporal variation was not confirmed in other studies [8,9,25,30,31]. Cats may enlarge their home ranges in spring in response to changes in the weather and available prey. It is possible that seasonal variation was already represented in the dataset we analysed, since the lowest temperature in the tracking period was −5.2 °C and the highest temperature was 26.9 °C (mean temperature = 11.9 °C). However, if the mean temperature was lower, weather conditions could have constrained the movements of cats in the study.

The analyses are limited to cats with home ranges < 40 ha, while observations from three cats were removed to fit the model. The reason that some cats roam far remains to be revealed, and the current data were insufficient for such an assessment.

Besides GPS and the questionnaire, the other data sources SDFE and the CORINE Land Cover project were used in characterising the habitats, and data on rainfall was retrieved from DMI. The use of SDFE and CORINE Land Cover project can generate uncertainty in the definition of area types, creating types that may not represent the local area that cats use. This can lead to the misclassification of habitats. Moreover, we assume that DMI’s estimates of rainfall at municipality level represent all addresses within the municipality. This may not always be the case, but due to the relatively large number of observations, this uncertainty is likely to be minor.

## 5. Conclusions

By combining data from GPS tracking and a questionnaire, the present study investigated the movement patterns of companion cats in Denmark. The time cats spent away from their owner’s property was associated with their sex, age, and access to nature areas. Their daily distance moved was affected by age and breed, and by whether they had access to nature areas and whether it rained. Home range size was associated with the age and sex of the cat and whether it had access to nature areas.

Although the majority of the cats involved in this study spent most of their time at home and were less active when it rained, our results indicate that many cats roam a lot. Due to the large individual variability in the movement patterns of Danish free-ranging companion cats, it is plausible that roaming cats pass through many gardens, potentially giving rise to disagreements between neighbours. Also, since roaming domestic cats are attracted to nature areas, they may pose a risk to vulnerable populations of rodents, bats, other small mammals, birds, and reptiles living in nature areas close to human settlements.

In light of our findings, owners of outdoor cats may choose to fence their properties to prevent their cats from escaping, although in some cases this may not be economically or practically feasible, and it may not be optimal for cat welfare. Alternatively, owners may reach out to people living in their neighbourhoods to improve acceptance of their roaming cats. In terms of the protection of wildlife in nearby natural areas, the owners may seek information about when wildlife is particularly vulnerable to cat predation, either at certain times of day or at certain times of year and aim to confine their cats during these periods.

## Figures and Tables

**Table 1:** Final model for percentage of time spent away from home base. The pseudo-R-squared of the model was 0.25. In comparison, the full model, including all variables, had a pseudo-R-squared of 0.30. The reference category (intercept) consisted of intact male cats in the age group 1–3 years with access to nature areas.

Variable	Level	Estimate	Standard Error	*z*-Value	Pr (>|*z*|)
(Intercept)		0.095	0.395	0.240	0.810
Sex	Intact female	−0.375	0.601	−0.623	0.533
	Neutered male	−0.989	0.408	−2.424	0.015
	Neutered female	−0.964	0.406	−2.373	0.018
Age group	4–7	0.013	0.202	0.063	0.950
	8–16	−0.473	0.218	−2.175	0.030
Access to Nature areas	No	−0.622	0.255	−2.438	0.015

**Table 2:** Final model for cats’ daily distance moved (m). The R-square of the model was 0.59. The median for the standardised within-group residuals was 0.031, with a minimum of −3.69, an IQR of −0.49 to 0.54 and a maximum of 3.57. The reference category (intercept) consisted of cats of mixed/unknown breeds in the age group 1–3 years with access to nature areas.

Variable	Level	Estimate	Standard Error	*t*-Value	*p*-Value
(Intercept)		16.590	0.673	24.658	<0.001
Rainfall (mm)		−0.045	0.018	−2.513	0.012
Access to Nature areas	No	−2.454	0.741	−3.310	0.001
Age group	4–7	−0.191	0.615	−0.311	0.757
	8–16	−2.463	0.625	−3.939	<0.001
Breed	Domestic shorthair	−1.228	0.656	−1.871	0.065
	Purebred	0.677	0.863	0.785	0.435

**Table 3:** Summary of predicted daily distance moved (m) at different levels of rainfall and 95% confidence intervals.

Rainfall (mm)	Mean	Lower 95%	Upper 95%
0	3564	2800	4453
5	3426	2683	4292
10	3291	2557	4151
15	3160	2425	4028
25	2908	2148	3826
30	2787	2008	3743
35	2670	1869	3669
45	2445	1599	3541

**Table 4:** Summary of sample sizes (*n*) and descriptive statistics for 95% BBKDE home range estimates stratified by the explanatory variables. SD = Standard deviation.

Variable	Level	*n*	Min.	1st Quartile	Median	Mean	3rd Quartile	Max.	SD
Overall	-	97	0.97	2.89	5.00	9.70	8.54	112.59	17.88
Sex	Neutered male	45	0.97	2.98	5.23	6.50	8.07	19.96	4.51
	Neutered female	45	1.01	1.96	4.44	12.62	8.54	112.59	25.19
	Intact male	4	3.79	9.03	15.72	18.01	24.70	36.80	14.32
	Intact female	3	1.97	2.00	2.04	2.94	3.42	4.80	1.61
Age group	1–3	32	0.97	3.69	7.39	17.94	15.91	112.59	29.00
	4–7	32	1.01	3.75	5.70	7.49	9.40	23.26	5.41
	8–16	33	1.02	1.61	2.57	3.86	5.25	11.62	2.82
Breed	Domestic shorthair	64	1.01	2.86	4.82	9.70	10.52	105.90	17.29
	Purebred	15	0.97	2.42	5.00	12.33	6.01	112.59	28.08
	Mixed/unknown	18	1.02	3.24	5.77	7.52	8.13	23.26	6.10
Access to	Ad libitum	79	0.97	2.31	4.96	10.24	8.74	112.59	19.67
Food	Restricted	18	1.79	4.43	6.04	7.32	7.64	17.60	4.75
Land use	Suburban/cottage	54	0.97	2.22	5.02	6.63	8.63	23.26	5.45
	Rural	31	1.28	3.77	5.60	16.80	11.20	112.59	29.74
	Urban/industry	12	1.20	2.39	3.43	5.20	5.92	19.21	4.99
Busy road	Yes	46	0.97	1.99	4.62	7.74	7.56	112.59	16.30
	No	51	1.01	3.25	5.60	11.47	11.20	105.90	19.18
Access to	Yes	81	1.01	3.45	5.60	10.84	10.78	112.59	19.29
Nature areas	No	16	0.97	1.58	2.21	3.94	4.40	19.21	4.48

**Table 5:** Final model for home range including variables Sex (sum of squares = 0.68), Age group (sum of squares = 1.40) and Access to Nature areas (sum of squares = 0.32). The estimates are given in log scale. The residual standard error of the model was 0.317, and the adjusted R-squared was 0.225. In comparison, the full model including all variables had a residual standard error of 0.317 and the adjusted R-squared was 0.226. The reference category (intercept) consisted of intact male cats in the age group 1–3 years with access to nature areas.

Variable	Level	Estimate	Standard Error	*t*-Value	Pr (>|*t*|)
(Intercept)		1.137	0.164	6.953	<0.001
Sex	Intact female	−0.551	0.244	−2.255	0.027
	Neutered male	−0.288	0.168	−1.714	0.090
	Neutered female	−0.365	0.168	−2.173	0.033
Age group	4–7	−0.029	0.081	−0.356	0.723
	8–16	−0.283	0.084	−3.382	0.001
Access to Nature areas	No	−0.163	0.091	−1.790	0.077

## Data Availability

Raw data resulting from the questionnaires are available as supplementary material to this publication. Data from GPS recordings are converted to BBKDE home range estimates for each cat; the raw data are not included, as the individual cat owner would be identifiable, which is not in line with data protection agreements.

## Supplementary Materials

The following supporting information can be downloaded at https://www.mdpi.com/article/10.3390/ani12141748/s1: Questionnaire for cat owners (S1), and file with raw, anonymised data and location errors of tracking devices (S2).

## Appendix A

Summary of individual cat information, tracking periods, and home range estimates:

**Cat**	**Sex**	**Age ^a^**	**Area**	**Municipality**	**Region**	**N ^b^**	**Days ^c^**	**Home Range ^d^**
01	Male	7	Suburban	Gribskov	Capital Region of Denmark	2344	7.0	8.95
02	Female	1	Suburban	Gribskov	Capital Region of Denmark	2447	7.0	3.52
03	Male	2	Cottage	Gribskov	Capital Region of Denmark	1775	6.9	10.89
04	Male	8	Cottage	Gribskov	Capital Region of Denmark	1309	7.0	7.68
05	Male	3	Cottage	Gribskov	Capital Region of Denmark	2031	7.0	5.03
06	Female	4	Cottage	Gribskov	Capital Region of Denmark	1794	7.0	10.96
07	Female	3	Cottage	Gribskov	Capital Region of Denmark	2402	7.0	4.44
08	Female	3	Cottage	Gribskov	Capital Region of Denmark	3248	9.6	13.32
09	Female	2	Rural	Gribskov	Capital Region of Denmark	2125	16.1	3.75
10	Male	9	Rural	Stevns	Region Zealand	548	6.6	1.28
11	Female	12	Industry	Gribskov	Capital Region of Denmark	1180	7.0	1.96
12	Male	13	Industry	Gribskov	Capital Region of Denmark	1555	7.1	1.20
13	Female	3	Rural	Gribskov	Capital Region of Denmark	1552	7.0	4.80
14	Male	13	Urban	Rødovre	Capital Region of Denmark	823	7.1	2.98
15	Female	9	Urban	Rødovre	Capital Region of Denmark	891	6.9	4.27
16	Male	8	Cottage	Gribskov	Capital Region of Denmark	1879	7.1	4.96
17	Female	4	Industry	Gribskov	Capital Region of Denmark	1697	7.0	19.21
18	Male	7	Urban	København	Capital Region of Denmark	1185	7.0	5.23
19	Male *	2	Rural	Furesø	Capital Region of Denmark	2717	7.1	36.80
20	Female	4	Rural	Thisted	North Denmark Region	2345	7.0	5.81
21	Male	7	Suburban	Greve	Region Zealand	2764	7.1	12.86
22	Female	12	Rural	Furesø	Capital Region of Denmark	1172	7.0	11.62
23	Female	12	Suburban	Horsens	Central Denmark Region	1063	6.4	2.57
24	Male	10	Rural	Halsnæs	Capital Region of Denmark	4081	7.1	4.26
25	Male	4	Rural	Sønderborg	Region of Southern Denmark	1995	7.0	5.15
26	Male *	1	Rural	Viborg	Central Denmark Region	1432	7.1	20.66
27	Female	4	Rural	Næstved	Region Zealand	1768	6.7	4.32
28	Male	13	Suburban	Albertslund	Capital Region of Denmark	1344	6.9	2.89
29	Female	12	Urban	København	Capital Region of Denmark	4381	6.0	2.33
30	Male	5	Suburban	Esbjerg	Region of Southern Denmark	1965	7.0	3.26
31	Female	4	Suburban	Ballerup	Capital Region of Denmark	1551	5.9	8.38
32	Male	11	Suburban	Fredensborg	Capital Region of Denmark	752	7.0	6.34
33	Male	10	Rural	Roskilde	Region Zealand	1021	7.0	8.28
34	Female	10	Suburban	Ballerup	Capital Region of Denmark	1028	7.0	1.37
35	Male	1	Rural	Faxe	Region Zealand	1237	7.0	17.60
36	Male	8	Suburban	Rudersdal	Capital Region of Denmark	813	7.0	2.05
37	Female	16	Suburban	Rudersdal	Capital Region of Denmark	651	7.0	1.02
38	Male	9	Suburban	Aarhus	Central Denmark Region	1316	7.0	7.70
39	Male	10	Rural	Gribskov	Capital Region of Denmark	1509	7.0	8.07
40	Male	12	Suburban	Kolding	Region of Southern Denmark	1184	7.0	1.76
41	Female	5	Rural	Slagelse	Region Zealand	1706	7.0	8.50
42	Male	4	Rural	Næstved	Region Zealand	1579	7.0	6.84
43	Female *	1	Suburban	Egedal	Capital Region of Denmark	756	10.5	8.96
44	Male	6	Suburban	Gribskov	Capital Region of Denmark	1395	5.9	5.28
45	Female	5	Urban	København	Capital Region of Denmark	1037	7.0	4.54
46	Male	6	Suburban	Køge	Region Zealand	1858	7.0	6.76
47	Female	2	Suburban	Roskilde	Region Zealand	2134	7.0	7.26
48	Male	3	Suburban	Faxe	Region Zealand	2306	7.1	6.53
49	Female	2	Industry	Aarhus	Central Denmark Region	1509	7.0	10.13
50	Male	15	Suburban	Gentofte	Capital Region of Denmark	284	7.0	1.85
51	Female	4	Rural	Aabenraa	Region of Southern Denmark	623	7.0	3.59
52	Female	4	Rural	Aabenraa	Region of Southern Denmark	788	7.0	5.60
53	Male	3	Suburban	Herning	Central Denmark Region	1105	7.0	19.96
54	Male	2	Suburban	Herning	Central Denmark Region	721	7.0	10.44
55	Female	4	Suburban	Herning	Central Denmark Region	690	7.0	6.05
56	Male	3	Suburban	Slagelse	Region Zealand	9023	7.0	2.97
57	Male	1	Suburban	Slagelse	Region Zealand	11085	7.0	2.10
58	Female	9	Suburban	Guldborgsund	Region Zealand	2390	7.0	1.64
59	Female	5	Suburban	Brønderslev	North Denmark Region	2437	7.0	23.26
60	Female	2	Suburban	Brønderslev	North Denmark Region	1961	7.1	6.32
61	Male	9	Urban	København	Central Denmark Region	678	6.8	11.45
62	Female	12	Rural	Sønderborg	Region of Southern Denmark	904	7.0	7.21
63	Female	7	Rural	Sønderborg	Region of Southern Denmark	464	7.0	3.62
64	Female	8	Rural	Sønderborg	Region of Southern Denmark	1044	7.0	4.83
65	Female	9	Suburban	København	Capital Region of Denmark	623	7.1	1.42
66	Female	3	Suburban	København	Capital Region of Denmark	667	7.1	1.86
67	Male	3	Suburban	København	Capital Region of Denmark	567	6.9	0.97
68	Male	1	Industry	Lolland	Region Zealand	2022	6.5	2.53
69	Female	1	Industry	Lolland	Region Zealand	1406	6.5	3.24
70	Female	10	Suburban	Aarhus	Central Denmark Region	716	7.1	1.51
71	Female	1	Rural	Vejle	Region of Southern Denmark	909	7.0	105.90
72	Female	1	Suburban	Aarhus	Central Denmark Region	8703	7.1	11.44
73	Male	9	Suburban	Køge	Region Zealand	1429	7.0	5.25
74	Male	11	Suburban	København	Capital Region of Denmark	422	7.1	1.79
75	Male *	4	Rural	Aabenraa	Region of Southern Denmark	1606	7.0	10.78
76	Male *	4	Rural	Aabenraa	Region of Southern Denmark	1233	7.0	3.79
77	Female	1	Rural	Vejle	Region of Southern Denmark	930	7.1	91.25
78	Female	1	Rural	Kalundborg	Region Zealand	898	7.0	112.59
79	Male	2	Suburban	Køge	Region Zealand	1811	7.0	15.56
80	Male	8	Suburban	Køge	Region Zealand	1435	7.2	5.00
81	Male	5	Suburban	Næstved	Region Zealand	880	7.0	6.27
82	Female	6	Suburban	Næstved	Region Zealand	464	7.0	3.45
83	Female	6	Suburban	Næstved	Region Zealand	834	7.0	20.88
84	Male	2	Suburban	Næstved	Region Zealand	815	7.0	7.51
85	Female	4	Suburban	Fredericia	Region of Southern Denmark	1161	7.0	1.01
86	Male	4	Suburban	Fredericia	Region of Southern Denmark	1280	7.0	3.22
87	Female	3	Suburban	Fredericia	Region of Southern Denmark	1814	7.0	1.74
88	Female	1	Suburban	Norddjurs	Central Denmark Region	675	7.0	16.97
89	Male	2	Suburban	Norddjurs	Central Denmark Region	589	7.0	13.99
90	Female *	8	Rural	Aabenraa	Region of Southern Denmark	857	7.0	2.04
91	Female *	6	Rural	Aabenraa	Region of Southern Denmark	905	7.0	1.97
92	Female	9	Rural	Vordingborg	Region Zealand	517	7.1	1.61
93	Male	7	Suburban	Køge	Region Zealand	1617	9.4	4.62
94	Male	7	Suburban	Assens	Region of Southern Denmark	2093	7.0	12.19
95	Male	7	Rural	Assens	Region of Southern Denmark	1347	7.0	4.75
96	Male	5	Rural	Assens	Region of Southern Denmark	508	6.9	12.12
97	Female	9	Rural	Aabenraa	Region of Southern Denmark	684	7.0	1.38
* Intact cat. ^a^ Age in years. ^b^ Number of recorded locations. ^c^ Tracking period in days. ^d^ 95% BBKDE home range estimates measured in hectares.								
