# Supplementary material for: Movement Patterns of Roaming Companion Cats in Denmark—A Study Based on GPS Tracking
Source: Animals (Basel). 2022 Jul 7;12(14):1748. doi: 10.3390/ani12141748 (PMC9311815; doi:10.3390/ani12141748)
Supplement: Supplementary file 1 [file animals-12-01748-s001.zip › Questionnaire (Supplementary S1).pdf]

Questionnaire for cat owners

Dear cat owner.

Thank you for your contribution to the study on cats' movement patterns!
The purpose of the study is to investigate the movement patterns of Danish free-ranging companion cats via GPS tracking, and to analyse whether variables such as the cats' sex, age, neuter status, and habitat influence their movement behaviour.

Therefore, we ask a series of questions regarding your cat, its surroundings and outdoor access. This information will shed light on why some cats range further than others.

The collected data is processed confidentially and is anonymised as soon as possible, i.e. when the data processing is complete. The study is conducted as a master's thesis in veterinary medicine and is submitted and evaluated in January 2022. The anonymised results of the study will subsequently be published in a scientific article.

You can withdraw from the study at any time until the data has been anonymised.

By answering the questionnaire, you hereby give your informed consent to the following:
- Your information is processed confidentially and included in the thesis.
- Your information may be shared with supervisors and any external examiner in connection with the assessment of the thesis.
- Your information may be published in an anonymised form in a scientific article.

Best regards,
Helene Ane Jensen

1. Please write the tracker ID which can be found on the back of the GPS or under 'Tracker' on the cat's profile in the Tractive app

____

2. Please write the date you received the GPS (dd/mm)

____

3. What is the name of the cat wearing the GPS?

____

4. Age of the cat

The cat is ______ years old

- ❑ Do not know

5. Which category best describes the cat? (1)

- ❑ A neutered female
- ❑ An intact female
- ❑ A neutered male
- ❑ An intact male
- ❑ Do not know

6. What is the breed of the cat?

Please write the breed of the cat here or choose one of the following options:

____

- ❑ Domestic shorthair
- ❑ Mixed breed
- ❑ Do not know

7. Which category best describes the cat? (2)

(1) ❑ Indoor cat with outdoor access via cat flap

(2) ❑ Indoor cat with outdoor access via window or door

(3) ❑ Indoor cat with outdoor access part of the year (e.g. in summer cottage)

(4) ❑ Outdoor cat or farm cat which is rarely or never indoors

(5) ❑ Non-socialised cat from a 'cat colony' or the like

(6) ❑ Other (please specify)

8. How is the cat's outdoor access best described? (Not asked if 7.4 is selected)

- ❑ The cat is able to go outside and inside as it pleases at all hours day and night
- ❑ The cat is typically let out during the day and is indoors at night
- ❑ The cat is typically let out at night and is indoors during the day
- ❑ Other (please specify)

9. Does the cat have *ad libitum* access to feed?

❑ Yes ❑ No

10. Are there any nature areas, forest areas or agricultural land within 1 kilometre of your home address?

❑ Yes ❑ No

11. Are there any busy roads within 300 metres of your home address?

❑ Yes ❑ No

Finally, please send a picture of the cat as well as the tracker ID (which can be found on the back of the GPS or under 'Tracker' in the Tractive app) to Helene Ane Jensen.

Thank you for your participation!
